# Fabrication of Poly-l-lactic Acid/Dicalcium Phosphate Dihydrate Composite Scaffolds with High Mechanical Strength—Implications for Bone Tissue Engineering

**DOI:** 10.3390/jfb6041036

**Published:** 2015-11-04

**Authors:** Nida Tanataweethum, Wai Ching Liu, W. Scott Goebel, Ding Li, Tien Min Chu

**Affiliations:** 1 Department of Biomedical Engineering, Indiana University-Purdue University Indianapolis, Indianapolis, IN 46202, USA; E-Mail: nidatana@umail.iu.edu; 2 Department of Biomedical and Applied Sciences, School of dentistry, Indiana University, Indianapolis, IN 46202, USA; E-Mails: criswcliu@gmail.com (W.C.L.); dli6@uky.edu (D.L.); 3 Department of Pediatrics, School of Medicine, Indiana University, Indianapolis, IN 46202, USA; E-Mail: sgoebel2@iu.edu

**Keywords:** calcium phosphate cement, scaffolds, mechanical properties, cytocompatibility, PLLA

## Abstract

Scaffolds were fabricated from poly-l-lactic acid (PLLA)/dicalcium phosphate dihydrate (DCPD) composite by indirect casting. Sodium citrate and PLLA were used to improve the mechanical properties of the DCPD scaffolds. The resulting PLLA/DCPD composite scaffold had increased diametral tensile strength and fracture energy when compared to DCPD only scaffolds (1.05 *vs.* 2.70 MPa and 2.53 *vs.* 12.67 N-mm, respectively). Sodium citrate alone accelerated the degradation rate by 1.5 times independent of PLLA. Cytocompatibility of all samples were evaluated using proliferation and differentiation parameters of dog-bone marrow stromal cells (dog-BMSCs). The results showed that viable dog-BMSCs attached well on both DCPD and PLLA/DCPD composite surfaces. In both DCPD and PLLA/DCPD conditioned medium, dog-BMSCs proliferated well and expressed alkaline phosphatase (ALP) activity indicating cell differentiation. These findings indicate that incorporating both sodium citrate and PLLA could effectively improve mechanical strength and biocompatibility without increasing the degradation time of calcium phosphate cement scaffolds for bone tissue engineering purposes.

## 1. Introduction

Bone grafts are commonly used in orthopedic surgeries to treat large bone defects caused particularly by trauma. Autologous bone graft is considered the “gold standard” among bone grafts because of its high efficiency in bone repair [1,2,3]. However, specific donor site morbidity and limited supply of material from an individual patient restrict its application [4,5]. Therefore, alternative strategies are urgently needed to repair bone defects. An ideal bone substitute will be a scaffold that provides a temporary three-dimensional (3D) guidance for cell attachment, growth and tissue regeneration. It is also essential for a bone scaffold to be biocompatible, biodegradable, and have mechanical strength [6].

Calcium phosphate (CaP) materials such as hydroxyapatite (HA) and β-tricalcium phosphate are the favored materials to fabricate bone scaffolds because their composition is similar to the mineral components in natural bones [7,8,9,10]. Besides, they were shown to be highly biocompatible and osteo-conductive because of their ability to support the growth of bone cells and build a strong bond between an implant and the peripheral tissues [3,11,12,13,14]. However, degradation of calcium phosphate crystals is slow mainly due to their large grain size and crystallinity that result from sintering during the manufacturing process. On the other hand, calcium phosphate cement is a self-setting alternative to calcium phosphate materials, and consists of micron-sized crystals that enhance their resorbability when compared to sintered ceramics [7]. Particularly, calcium phosphate cements composed of dicalcium phosphate dehydrate cements (DCPD) show excellent degradability and high resorbability, and accelerate formation of a new bone surface [11,15,16]. In addition, DCPDs are also highly moldable and injectable due to their consistent paste-like nature and ability to set at room temperature [17]. These major advantages not only benefit during the filling of bone defects but are also important for the fabrication of 3D bone scaffolds [18]. The main limitations of DCPD scaffolds are their brittleness and poor mechanical strength.

Previous studies have established that mechanical properties of inorganic scaffolds could be improved by modifying setting conditions and preparing polymer composites [19,20,21]. Thus, higher cement compaction was achieved in DCPD scaffolds by optimizing DCPD setting conditions using regulators that inhibited crystal growth and reduced the average crystal size [22]. In addition, mechanical properties of the DCPD scaffolds were increased by adding pyrophosphate salts and α-hydroxyl carboxylic acids (and their salts), such as citric acid, tartaric acid, and sodium citrate that raised the cement powder to liquid ratio [23,24,25]. We previously showed that 100 mM sodium citrate best improved the compressive strength of DCPD scaffold from 1 to 2.0 MPa [25].

Scaffolds fabricated from a single material more often have limited biological performance thus the use of composite scaffolds have been actively sought after. Composite scaffolds containing calcium phosphates can be effectively fabricated by immersing a calcium phosphate cement scaffold into a polymer solution that coats a thin layer of polymer [10,20], which improves the brittle and porous calcium phosphate. This approach also fills the micro-cracks and struts with the polymer thus strengthening the calcium phosphate scaffolds, which are widely applied in bone tissue engineering [26]. Potential coating materials to fabricate composite scaffolds include poly hydroxy esters, such as polycaprolactone (PCL), poly-l-lactic acid (PLLA), and poly glycolic acid-co-lactic acid (PLGA), which are currently used as biodegradable polymers [26,27,28]. PLLA is a synthetic polymer and has excellent biocompatibility. For example, osteoblast proliferation was shown to be high on PLLA coated HA specimens compared to uncoated specimens, and the mechanical strength of PLLA coated HA specimens was 50–300 times higher when compared to uncoated specimens [26]. In addition, proliferation and adherence of human bone marrow derived mesenchymal stem cells was enhanced on nano-composite HA/PLLA coated BCP scaffolds [26]. However, PLLA has a low degradation rate and only 50% crystallinity, which is lower than other synthetic polyesters, such as poly(lactic-*co*-glycolic)acid and polyglycolic acid [29]. Furthermore, inflammatory macrophages and multinucleated giant cells were found on the surface of PLLA screws implanted in sheep bones [30].

In a previous study, we developed a novel method termed indirect casting to fabricate precisely controlled scaffold structure according to a CAD model [18]. The CAD model was applied to control the exact pore size, location, and interconnectivity of the scaffolds. Poly (propylene fumarate) was used to reinforce DCPD scaffolds to improve the mechanical properties [21]. The resulting composite scaffold had a 26-fold increase in compressive strength although the degradation of these scaffolds was slow. More importantly, inflammation was observed *in vivo* likely due to incomplete polymerization. In our pursuit to fabricate a better composite scaffold for bone tissue engineering, in this study we developed a PLLA/DCPD composite scaffold with enhanced initial compressive strength and toughness using a setting regulator and polymer coating. Characterization of these scaffolds showed improved mechanical strength, high degradation rate and cytocompatibility of the DCPD/PLLA composite scaffolds indicating their potential to be used for bone tissue engineering.

## 2. Results

### 2.1. Incorporation of PLLA on DCPD Disks

The range of PLLA mass incorporated in all PLLA/DCPD composite disks was 0.05–0.06 mg/mm^3^ (Table 1). Variations in the P/L mass ratio and liquid components did not significantly affect PLLA incorporation (*p* > 0.05).

**Table 1:** Amount of PLLA incorporated into DCPD disks at various P/L ratio.

P/L Ratio	Liquid Phase	PLLA Incorporation (mg/mm^3^)
1.00	Deionized Water	0.06 ± 0.01
	Sodium Citrate	0.05 ± 0.01
1.25	Deionized Water	0.06 ± 0.01
	Sodium Citrate	0.05 ± 0.01
1.50	Deionized Water	0.06 ± 0.01
	Sodium Citrate	0.05 ± 0.01

### 2.2. Diametral Tensile Strength

**Figure 1:**
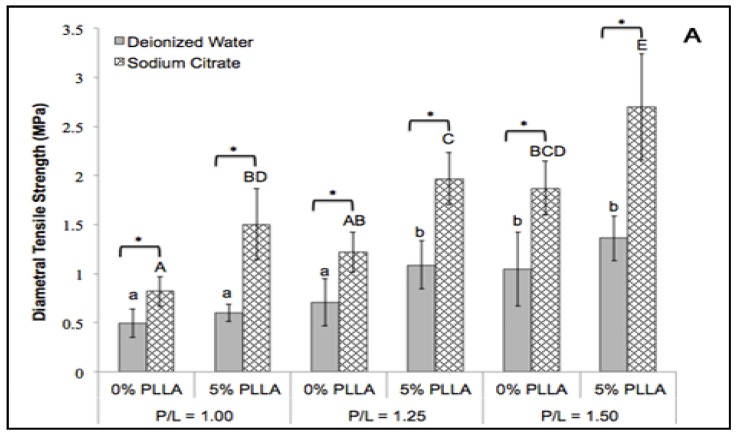

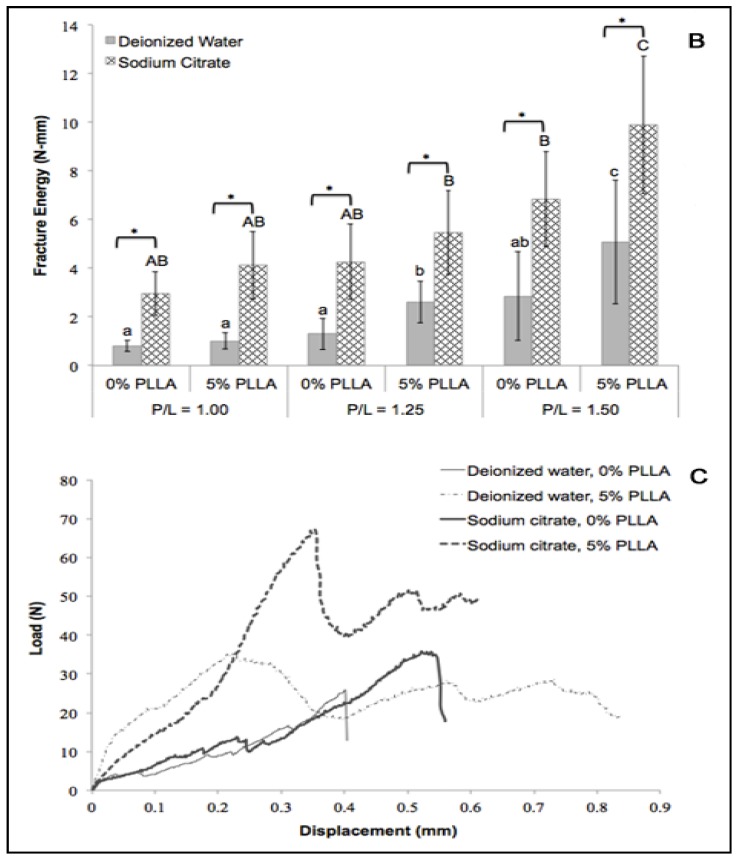
Diametral compression testing. Diametral tensile strength (**A**); Fracture energy (**B**) and Load-displacement curve (**C**) obtained from DCPD (0% PLLA) or DCPD/PLLA composite (5% PLLA) at the P/L 1.50 ratio. Different lower case letters and upper case letters indicate significant differences within the deionized water or sodium citrate groups, respectively (*n* = 10; *p* < 0.05). * represent significant differences between the indicated groups (*n* = 10; *p* < 0.05).

Sodium citrate significantly increased the diametral tensile strength of the DCPD disks at all P/L mass ratios (Figure 1A) (*p* < 0.05). The greatest improvement was found at P/L 1.50 ratio. The diametral tensile strength of the DCPD only group increased from 1.05 ± 0.37 MPa to 1.87 ± 0.27 MPa. In comparison, PLLA coating significantly increased the diametral tensile strength of the DCPD group prepared from sodium citrate in all P/L ratios (*p* < 0.05). However, the same effect was only observed at P/L 1.25 for the DCPD disks prepared from deionized water. The highest diametral tensile strength was 2.70 ± 0.54 MPa found in the PLLA/DCPD composite group prepared from sodium citrate at P/L 1.50 ratio. The synergistic effect of PLLA coating and sodium citrate in improving the diametral tensile strength (2.58-fold increase) of the DCPD group was observed at P/L 1.50 ratio (Figure 1A).

### 2.3. Fracture Energy

The use of sodium citrate as the setting regulator significantly improved the fracture energy of the DCPD disks at all P/L ratios (Figure 1B) (*p* < 0.05). The greatest improvement in fracture energy was found at the P/L 1.50 ratio. Fracture energy of the DCPD only group increased from 2.53 ± 1.62 MPa to 6.31 ± 1.05 N-mm. PLLA coating significantly increased the fracture energy of DCPD prepared from deionized water at P/L 1.25 and 1.50 ratios (*p* < 0.05). However, the same effect was only observed at P/L 1.25 ratio for DCPD prepared from sodium citrate. The highest fracture energy was 12.67 ± 4.97 N-mm found in the PLLA/DCPD composite prepared from sodium citrate at the P/L 1.50 ratio. Together, PLLA coating and sodium citrate improved the fracture energy of DCPD at P/L 1.50 three-fold thus indicating their synergistic action.

Figure 1C shows typical diametral compressive load-displacement curves obtained during diametral compression tests for DCPD and PLLA/DCPD composite groups at the P/L 1.50 ratio. All four curves demonstrated a linear elastic behavior at the beginning of the experiment. However, after failure the loads of DCPD only specimens sharply dropped but the loads of PLLA/DCPD composites remained unchanged with increase in time. These results indicate the higher yield resistance of PLLA/DCPD composites than the DCPD only group.

### 2.4. In Vitro Degradation Behavior

**Figure 2:**
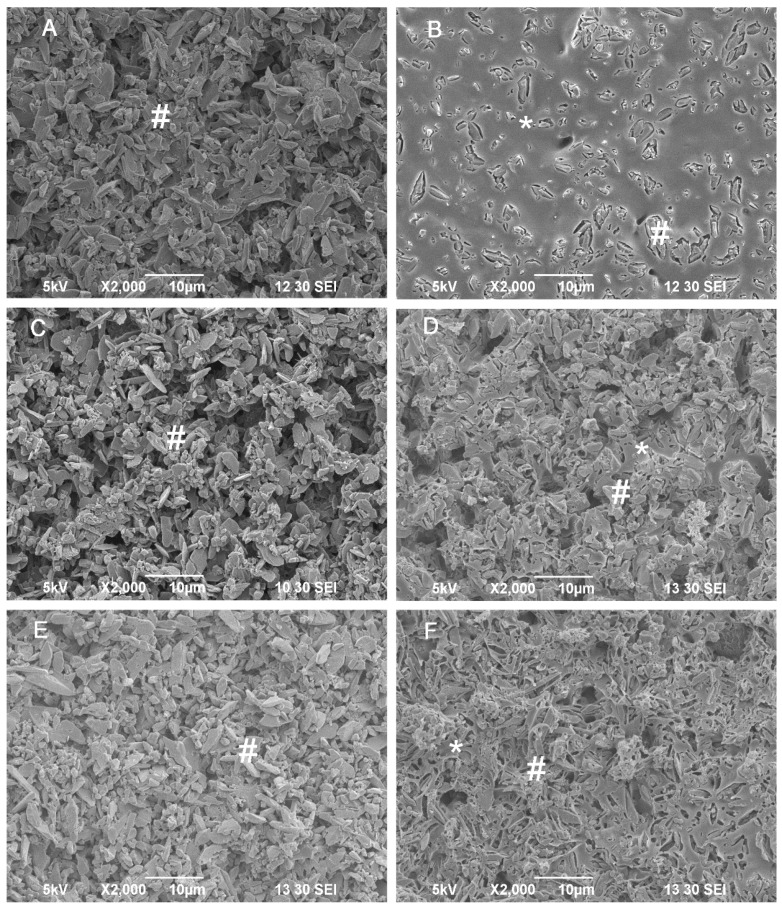
SEM images of the DCPD (A,C,E) and DCPD/PLLA composite (B,D,F) prepared from deionized water and immersed in PBS for 7 days (**A**,**B**), 28 days (**C**,**D**) or 56 days (**E**,**F**). # and * indicate DCPD crystals and PLLA coating, respectively.

**Figure 3:**
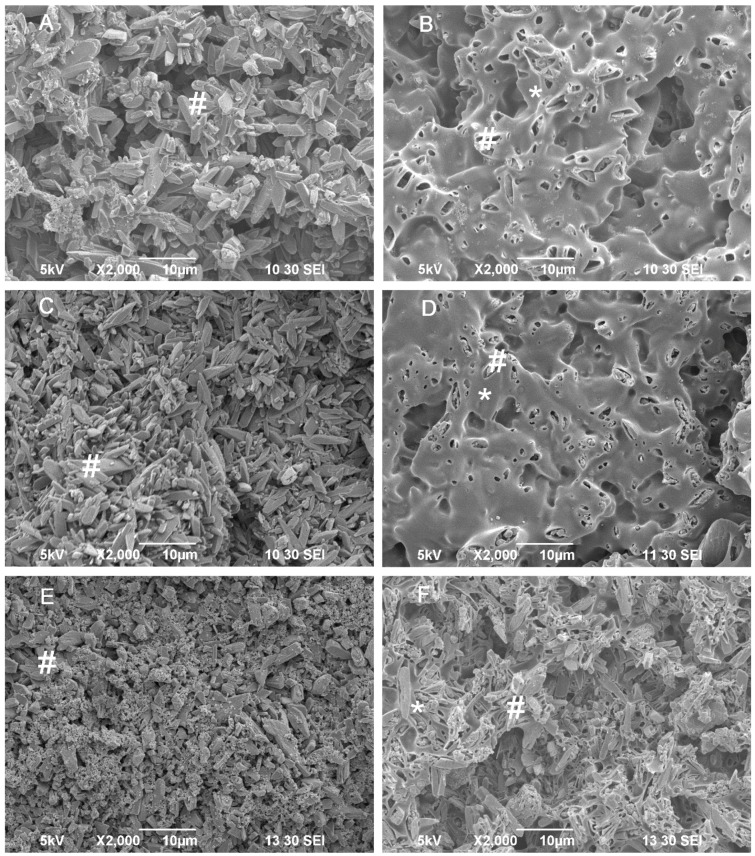
SEM images of DCPD (A,C,E) and DCPD/PLLA composite (B,D,F) prepared from sodium citrate and immersed in PBS for 7 days (**A**,**B**), 28 days (**C**,**D**) or 56 days (**E**,**F**). # and * indicate DCPD crystals and PLLA coating, respectively.

DCPD crystals prepared from sodium citrate had finer crystalline structures than DCPD crystals prepared from deionized water (Figure 2A and Figure 3A). PLLA coating on the DCPD matrix was clearly visible (Figure 2B and Figure 3B). After immersion of the PLLA/DCPD composite in PBS for 28 days, the PLLA coat on DCPD gradually degraded exposing more DCPD matrix to the solution. After immersion for 56 days, most of the PLLA coat had degraded thus exposing most of the DCPD matrix. However, a certain amount of PLLA that still remained on the DCPD surface maintained the specimen integrity. On the other hand, the crystal structures of DCPD formed from deionized water and sodium citrate on day 56 were smaller than the crystals formed on day 7.

**Figure 4:**
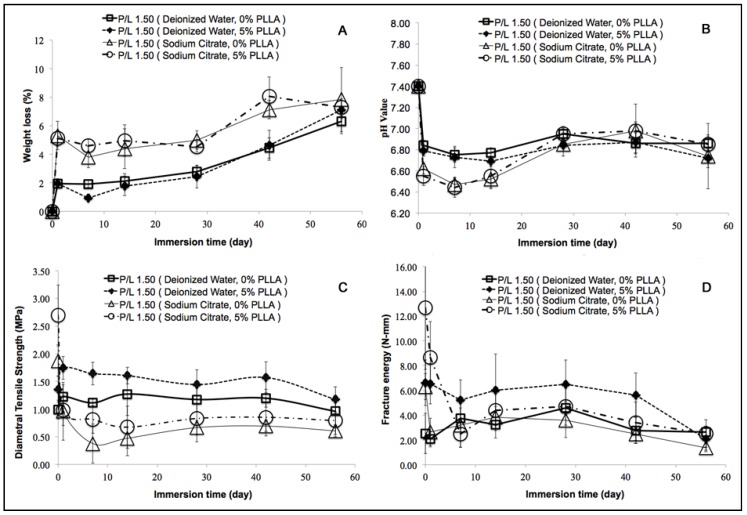
Weight loss (**A**); pH value (**B**); diametral tensile strength (**C**); and fracture energy (**D**) of DCPD (0% PLLA) and PLLA/DCPD composite (5% PLLA) during *in vitro* degradation.

Measurement of the DCPD and PLLA/DCPD disk weight loss (%) *in vitro* indicated a gradual increase in weight loss with an increase in the immersion time in PBS (Figure 4A). After 56 days, the weight loss (%) of both DCPD and PLLA/DCPD groups prepared from deionized water was significantly higher than the weight loss after 14 days (*p* < 0.05). The weight loss of both groups significantly increased from 2.13% ± 0.52% to 6.32% ± 0.88% and 1.78% ± 0.69% to 7.12% ± 0.64%, respectively (*p* < 0.05) from day 14 to day 56. From day 1 to day 42, both DCPD and PLLA/DCPD groups prepared from sodium citrate showed significantly higher weight loss compared to the respective groups prepared from deionized water (*p* < 0.05). However, PLLA coating did not have any significant effect on weight loss *in vitro* regardless of whether DCPD and PLLA/DCPD were prepared from deionized water or sodium citrate.

The pH values of PBS in both DCPD and PLLA/DCPD samples dropped sharply after immersion for one day (Figure 4B). After seven days, there was a gradual increase in pH values, which however decreased again after 42 days. Finally, the pH values of all sample groups were between pH 6.4 and 7.0 after 56 days. Interestingly, the pH values were not significantly different among these groups at the end point of 56 days. The lowest pH values were 6.47 ± 0.07 and 6.44 ± 0.09 found on day 7 in both DCPD and PLLA/DCPD groups prepared from sodium citrate, respectively.

After immersion in PBS for 7 days, diametral tensile strength and fracture energy of both DCPD and PLLA/DCPD composite groups decreased sharply (Figure 4C,D). These parameters stabilized after day 7 but declined again after 42 days. The PLLA/DCPD composite groups prepared from deionized water and sodium citrate had higher diametral tensile strength and fracture energy than the DCPD without PLLA coating. Both DCPD and PLLA/DCPD groups prepared from sodium citrate had higher diametral tensile strength and fracture energy at the starting time point but lower fracture energy after immersion in PBS for 56 days when compared to their counterparts prepared from deionized water. In addition, DCPD prepared from sodium citrate had the lowest diametral tensile strength and fracture energy during degradation.

### 2.5. Mechanical Property of the Composite Scaffolds

**Figure 5:**
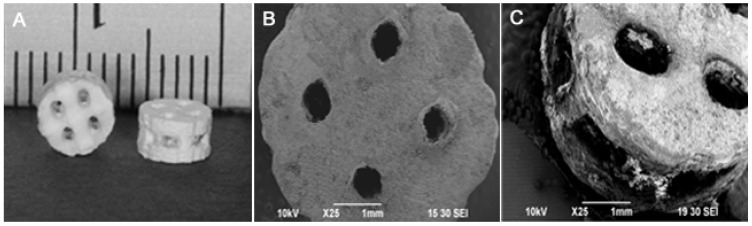
Digital image of the DCPD scaffold (**A**); SEM image representing a macroscopic aerial view of the scaffold (**B**); SEM image representing a macroscopic lateral view of the scaffold (**C**).

**Figure 6:**
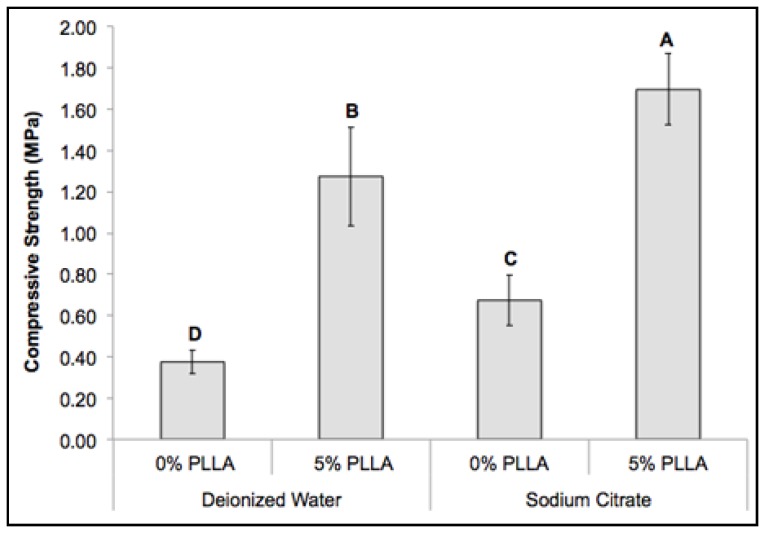
Compressive strength of DCPD (0% PLLA) and PLLA/DCPD composite scaffolds (5% PLLA) at the 1.50 P/L molar ratio. Different letters above bars represent significant differences among the various scaffolds (*n* = 6; *p* < 0.05).

A thin layer of PLLA coating was applied to improve the mechanical property of the DCPD scaffolds, which contained intersecting rectangular beams. The scaffolds were fabricated using an indirect casting method to control the scaffold structure (Figure 5). Scaffold fabrication with sodium citrate significantly improved the compressive strengths of both DCPD and PLLA/DCPD composite scaffolds (*p* < 0.05) (Figure 6). The compressive strength of DCPD scaffolds increased from 0.37 ± 0.06 MPa to 0.67 ± 0.12 MPa (*p* < 0.05). In addition, the PLLA coating also had a significant effect on the compressive strength of DCPD only groups prepared from deionized water and sodium citrate. The greatest increase in the compressive strength from 0.37 ± 0.06 MPa to 1.27 ± 0.24 MPa (*p* < 0.05) was observed in the PLLA coated DCPD group prepared from deionized water. The highest compressive strength of 1.70 ± 0.17 MPa was recorded in the PLLA/DCPD composite group prepared from sodium citrate.

### 2.6. Cytocompatibility of the DCPD Cements and Cell Proliferation

**Figure 7:**
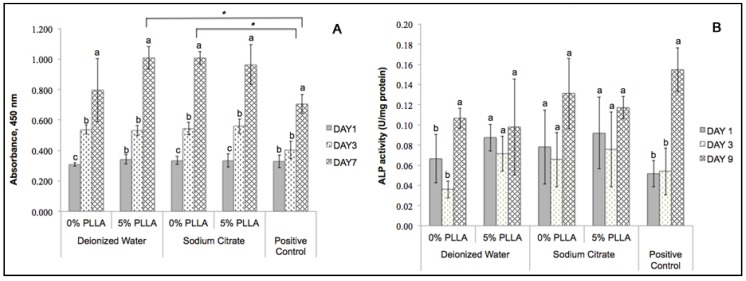
Cell viability (**A**) and ALP activities (**B**) of dog-BMSCs cultured in growth medium conditioned with DCPD (0% PLLA) or PLLA/DCPD composite (5% PLLA). The standard medium was used as the positive control. Different letters above bars indicate significant differences within each DCPD and PLLA/DCPD composite. * indicates significant differences among the indicated groups (*n* = 3; *p* < 0.05).

XTT assay was used to investigate the cytocompatibility of the DCPD disks by determining the viability of dog-BMSCs grown in a DCPD conditioned medium (Figure 7A). The results showed that cell proliferation increased significantly from day 1 to day 7. In the PLLA/DCPD composites, PLLA coating likely deterred the quick release of DCPD into the culture medium. Preparation in deionized water or sodium citrate did not significantly affect cytocompatibility of the DCPD and PLLA/DCPD composite groups. In addition, cytocompatibility of DCPD prepared from sodium citrate and PLLA/DCPD composite prepared from deionized water were significantly higher than the positive control on day 7.

### 2.7. Determining Cell Differentiation by ALP Activity

Cell differentiation was assessed by the extent of ALP activity in dog-BMSCs cultured in DCPD or PLLA/DCPD conditioned osteogenic medium (Figure 7B). The pattern of ALP activity was similar in all groups with no significant difference in ALP activity among the various groups and the positive control. These results indicate that the cells were able to differentiate in the osteogenic medium conditioned with DCPD and PLLA/DCPD.

### 2.8. Cell Attachment and Morphology

**Figure 8:**
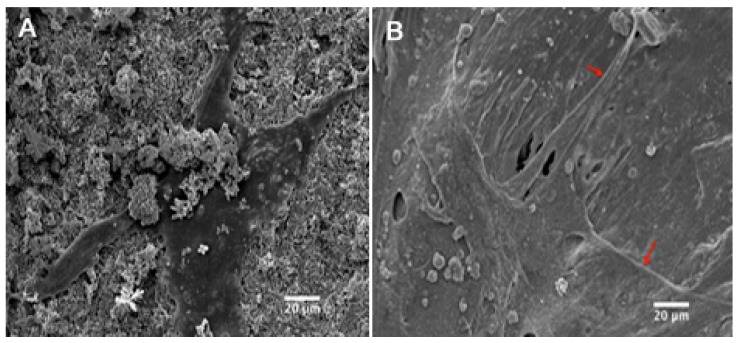
SEM image (1000×) of dog-BMSCs spread well on DCPD surface (**A**) and PLLA/DCPD composite surface (**B**).

SEM was applied to observe the morphology of dog-BMSCs on the surface of the DCPD and PLLA/DCPD composite (Figure 8). Significant differences were not observed in the number of cells that attached to the two surfaces after 7 days in culture. The cells generally attached on both groups and showed a well-spread morphology with a spread cytoskeleton.

## 3. Discussion

Effective bone repair requires scaffolds with highly interconnected macro pores and good mechanical properties that induce new bone tissue growth and improve load bearing. In the past, calcium phosphate cements have been widely used as scaffold material for orthopedic and craniofacial bone regeneration due to its excellent bioresorbability and osteoconductivity [31]. However, their low mechanical strength limits their use in load-bearing applications [17].

Previously, we developed an indirect casting with rapid prototyping technology to prepare DCPD scaffolds with completely interconnected pores and high compressive strength [18]. Since DCPD cement is weak and brittle, pure DCPD scaffolds would easily collapse *in vivo* due to their low mechanical strength. In this study, we applied the same indirect casting but included a polymer coating process to improve the tensile strength and toughness of the DCPD scaffolds. In addition, ceramic is only used, as filler and there is no chemical binding between polymer and inorganic material [32]. Therefore, this method could be applied to reinforce preset cement structures such as indirect casting or 3D printing [33].

Our results demonstrated that diametral tensile strength of the DCPD/PLLA composite (2.70 MPa) was comparable to cancellous bone with a tensile strength of 1–5 MPa [17,34]. In addition, combining sodium citrate (setting regulator) and PLLA coating significantly improved the diametral tensile strength, fracture energy, and compressive strength (Figure 1). Sodium citrate has been shown to improve the diametral tensile strength and compressive strength of DCPD cements by inhibiting DCPD crystal growth, which increased the compaction of the cement thus enhancing the mechanical strength of the cements [17,22]. Fracture-energy during compression tests was also higher for the PLLA coated samples than the uncoated samples. This finding supports our hypothesis that PLLA coating improves polymer bridges, which strengthens the samples and avoids cracks that develop in calcium phosphate cements. In general, calcium phosphate cement scaffolds contain open micro cracks that develop in the struts after dehydration. The thin layer of PLLA coating likely filled the pre-existing open micro pores and sealed micro cracks on the surface of the scaffolds to form an interlaced structure. This structure and the layer of polymer can hinder the formation of new cracks and strengthen DCPD cements. Such infiltration of polymers into calcium phosphate structures for strengthening purposes has been shown by a number of studies. Lei *et al.*, reported that PLLA/HA coating substantially improved the compressive strength of biphasic calcium phosphate scaffolds from 0.31 to 3.95 MPa [35]. In addition, PLA infiltration improved the flexural strength of B-TCP scaffolds by a factor of five [36]. Similarly, polymer ligaments stretched on the crack openings increased energy consumption during the propagation of micro cracks [37]. In humans, the collagen fibril has been shown to maintain bone tissue toughness by bridging the cracks [37]. In our study, PLLA acted in a fashion similar to natural collagen fibrils in the human body and increased the toughness of the DCPD/PLLA composite.

Further, we investigated the *in vitro* degradation of the DCPD and PLLA/DCPD composite in PBS at 37 °C by characterizing the weight loss, pH value, diametral tensile strength, fracture energy, and morphology of the crystals. Our results revealed that the pH value of all groups declined initially until day 7 followed by an increase till day 42 and a further decrease beyond this time. The degradation of DCPD occurs in three steps: dissolution, disintegration, and precipitation [38,39] and is affected by the concentration of calcium ions in the medium [40]. The dissolution of DCPD likely initiated when DCPD was placed in PBS leading to DCPD weight loss as described in the following equation.

(1) $${\text{CaHPO}}_{4}\cdot 2H_{2}O\to {\text{Ca}}^{2+}+{\text{HPO}}_{4}^{2-}+2H_{2}O$$

As DCPD dissolution and disintegration increased hydroxyapatitie (HA) is formed according to the following equation.

(2) $$10{\text{CaHPO}}_{4}\cdot 2H_{2}O\to {\text{Ca}}_{10}{\left({\text{PO}}_{4}\right)}_{6}{\left(\text{OH}\right)}_{2}+4H_{3}{\text{PO}}_{4}+18H_{2}O$$

This reaction likely occurred because DCPD is metastable at pH values higher than 4 while HA is stable at pH higher than 4 [38,39]. It is plausible that formation of phosphoric acid (H_3_PO_4_) may have reduced the pH of PBS thus increasing the DCPD weight loss, which in turn decreased the fracture energy of the samples.

We have shown that sodium citrate could accelerate the degradation rate of DCPD cements (Figure 4A) and decrease the pH (Figure 4B). We used sodium citrate as the setting regulator to reduce the size of DCPD crystals [17]. It is likely that the fine crystal structure of DCPD increased the surface area of DCPD thus facilitating higher degradation. As a result, DCPD and PLLA/DCPD groups prepared from sodium citrate had higher weight loss and lower pH during degradation.

The degradation of PLLA at the early stage could have created a number of small pores in the PLLA film although the PLLA coating did not adversely affect the pH of the DCPD during degradation.

The diametral tensile strength and fracture energy corresponded well to the weight loss of the samples. During degradation, PLLA/DCPD composite from both deionized water and sodium citrate groups had higher diametral tensile strength and fracture energy than others without PLLA coating. It is likely that PLLA coating filled the pre-existing open micro pores and sealed the micro cracks to strengthen the DCPD cements and thereby slowed their degradation rate. However, the diametral tensile strength and fracture energy of both DCPD and PLLA/DCPD composite decreased as the degradation time increased. It is presumed that the mechanical strength of the scaffolds will be significantly enhanced once new bone starts to grow into the macro-pores of the scaffold after implantation. Therefore, mechanical strength of the scaffold is important only in the early stage of implantation.

Lastly, we investigated the cytocompatibility of DCPD and PLLA/DCPD composite by studying the attachment, proliferation, and differentiation of dog-BMSCs. Both DCPD and PLLA/DCPD stimulated dog-BMSCs attachment, proliferation, and differentiation thus indicating its safety in mammalian system. These results suggest that DCPD coated with PLLA could support the adhesion and growth of dog-BMSCs. This is consistent with a previous finding that PDLLA coating improved the spreading and viability of human bone-derived cells on CaSiO_3_ scaffolds [41]. The concentration of free calcium ion, phosphate ion and pH in the medium could have played a major role in the cytocompatability of DCPD and PLLA/DCPD composite. Previously, osteo-conductive properties of an electrospun scaffold were reported to improve by coating a layer of calcium phosphate. Calcium and phosphate released from the coating increased ALP expression, which lead to osteogenic differentiation [42].

## 4. Experimental Section

### 4.1. DCPD Disk Preparation

DCPD cement was prepared by mixing monocalcium phosphate monohydrate (MCPM; Strem Chemicals, Newburyport, MA, USA) and β-tricalcium phosphate (β-TCP; Fluka Chemical corporation, Ronkonkoma, NY, USA) at a molar ratio of 1:1. Deionized water and 100 mM sodium citrate were used as mixing liquids. The cement pastes were prepared with various ratios (1.00, 1.25, and 1.50) of powder to liquid mass (P/L). Finally, the DCPD disks (5 mm diameter and 2.5 mm thickness) were fabricated by casting the cement paste into appropriate sized molds. After allowing the cements to set for 10 min, they were removed from the mold and dried in a desiccator for 48 h. To fabricate the PLLA/DCPD composites, PLLA pellets (*M*_w_ = 105 kDa) were first dissolved in chloroform (CHCl_3_) to obtain a 5% (w/v) solution. Then, the DCPD disks were immersed in the PLLA solution under vacuum to allow PLLA infiltration into the porous structures. The disks were then removed from the solution, dried in a fume hood for 30 min and dried in the desiccator overnight to remove all the organic solvent.

### 4.2. Mechanical Testing and Degradation of Disk Specimens

The diametral tensile strength and fracture energy of DCPD and PLLA/DCPD composite scaffolds (5 mm diameter and 2.5 mm thickness) were measured using a universal material testing machine (MTS Systems, Eden Prarie, MN, USA). The specimens were loaded in diametral compression at a rate of 1 mm/min. Ten disks from each group were tested and the average value was calculated. The diametral tensile strength was determined based on the following equation:

$$\sigma_{\text{strength}}=2P/\pi DL\;$$

where “*P*” (N) is the failure load, “*D*” (m) is the diameter of specimen, and “*L*” (m) is the thickness of the specimen. Fracture energy was calculated from the load–displacement curve, compressive strength of the scaffolds was measured using MTS under the same condition as mentioned above. Each measurement was repeated five times/group.

### 4.3. In Vitro Degradation of Disk Specimens

*In vitro* degradation of DCPD and PLLA/DCPD specimens were performed in phosphate buffered saline (PBS) pH 7.4 at 37 °C. For this assay, we used only the disks fabricated at a 1.5 P/L ratio because the highest diametral tensile strength and fracture energy were found at this ratio. To monitor the change in pH of the PBS and weight loss of the samples, several disks from each experimental group were placed in 3 mL PBS in individual glass vials and incubated at 37 °C for 56 days. PBS in the vials was refreshed every 14 days. Five specimens from each group were collected on days 1, 7, 14, 28, 42, and 56 followed by measuring the pH of the PBS in a pH meter (Denver Instruments, Arvada, CO, USA). Then, the disks were dried under vacuum in a desiccator chamber for 48 h. The fracture energy, weight loss (WL) and diametral tensile strength of these dried samples were measured under diametral compression. WL was calculated based on the following equation:

WL (%) = (*W*_0_ − *W*_d_)/*W*_0_ × 100%

where “*W*_0_” (g) is the initial weight of the specimens and “*W*_d_” (g) is the weight of the dried specimens at each time point. In addition, the surface of the specimens was evaluated using a scanning electron microscope (SEM) (JEOL JSM-5310LV, Peabody, MA, USA).

### 4.4. Scaffold Fabrication and Mechanical Testing

The CAD software (Rhinoceros McNeel North America, Seattle, WA, USA) was applied to design a cylindrical calcium phosphate scaffold (5 mm diameter × 2.77 mm thickness) containing 1 mm × 5 mm × 2. 77 mm rectangular beams spaced 800 μm apart. The macroporosity of the design was 72.72% [18]. Negative wax molds of the scaffolds were produced using a Solidscape T66 benchtop rapid prototyping machine (Solid-scape, Merrimack, NH, USA). DCPD cement paste was prepared at the P/L 1.50 ratio; DCPD scaffolds were fabricated by pressing the wax molds into the cement paste. After allowing the cement to set for 30 min the wax molds were dissolved in acetone to obtain the DCPD scaffolds. The PLLA/DCPD composite scaffolds were fabricated using the same protocol described above for the PLLA/DCPD composite disk specimen preparation.

### 4.5. In Vitro Cellular Study

Disks from the two groups (DCPD and PLLA/DCPD) were soaked in PBS for four days and the PBS was refreshed every 24 h to remove free MCPM. Then, they were sterilized by soaking in 70% ethanol for 30 min and subsequently washed twice in sterile PBS.

For the indirect cytocompatibility test, each disk was soaked in 1.6 mL of Dulbecco’s Modified Eagles Medium (DMEM; Invitrogen, Carlsbad, CA, USA) supplemented with 10% fetal bovine serum (FBS; Atlanta Biologicals, Lawrenceville, GA, USA), 1% antibiotic-antimycotic solution (Sigma-Aldrich, St. Louis, MO, USA), and 10 mM HEPES (Sigma-Aldrich, St. Louis, MO, USA). The medium was refreshed every two days. Cell proliferation and differentiation were evaluated by XTT and ALP assays as previously described [21]. To evaluate cell proliferation, dog-BMSCs were harvested from dog femurs [10] and ~2000 cells/well were plated on a 96-well plate. After allowing the cells to attach overnight at 37 °C they were cultured in the medium conditioned with DCPD or PLLA/DCPD disks for 24 h. The medium was refreshed every two days to remove dead cells and wastes. XTT sodium salt (2,3-Bis(2-methoxy-4-nitro-5-sulfophenyl)-2*H*-tetrazolium-5-carboxanilide inner salt, Sigma-Aldrich, St. Louis, MO, USA) was used to evaluate cell proliferation on days 1, 3, and 7. Corresponding changes in the absorbance of dye were measured photometrically at 450 nm.

To evaluate alkaline phosphatase (ALP) activity, ~5000 dog-BMSCs were plated per well in a 96-well plate. After allowing cells to attach to the plate surface they were grown in osteogenic medium (DMEM supplemented with the osteogenic factors, 10−8 M dexamethansone, 5 μg/mL ascorbic acid, and 20 mM β-glycerophosphate) that was previously conditioned by soaking the DCPD or PLLA/DCPD disks for 24 h. ALP activity was measured on days 3, 7, and 9 with the ALP assay kit (Sigma-Aldrich, St. Louis, MO, USA). At each time point, the cells were washed twice in PBS and lysed in 100 μL of 0.2% Triton X-100. Then, the cell lysates were subjected to three freeze/thaw cycles (−80/37 °C) for a total of 60 min. Cell lysates were placed in a 96-well plate to measure ALP activity according to the manufacturer’s instructions. Fluorescence was measured using a fluorometer (SpectraMax, Molecular Devices) at 360 nm excitation and 440 nm emission. ALP activity was normalized by total protein concentration determined using the BCA protein assay kit (Thermo Scientific Pierce, Rockford, IL, USA) and represented as units/gram of protein.

To investigate cell morphology and cell attachment on DCPD and PLLA/DCPD composites, two sample groups (DCPD and PLLA/DCPD composite from sodium citrate) were chosen for this study. Each disk was seeded with ~2000 dog-BMSCs in a 96-well plate and incubated in 100 μL culture medium for seven days. To visualize cell attachment, the disks were observed through an optical microscope after staining with 0.25% (w/v) neutral red (Sigma-Aldrich, St. Louis, MO, USA). Cell morphology on the surface of DCPD and the PLLA/DCPD composite were observed by SEM after fixing the samples in 10% v/v formaldehyde. Then, they were dehydrated in graded ethanol and hexamethyldisilizane (HDMS) solution, and sputter coated with gold prior to SEM imaging.

### 4.6. Statistical Analysis

Statistical analyses were performed with SPSS 20 (SPSS Inc, Chicago, IL, USA). One-way and two-way analysis of variance (ANOVA) with the post hoc Tukey-Kramer multiple-range tests were performed to determine the statistical significance of the differences among two or more groups. Significance level was set at *p* < 0.05.

## 5. Conclusions

In this study, we have shown that completely interconnected polymer/calcium phosphate cement scaffolds with significantly improved mechanical properties can be fabricated by combining indirect casting and polymer impregnation. Our findings also revealed that sodium citrate not only increased both compressive and diametral tensile strengths but also accelerated the degradation rate of DCPD cements. Cytocompatibility tests showed that DCPD and PLLA/DCPD composite were non-cytotoxic. Furthermore, they stimulated cell proliferation and differentiation. Lastly, this study showed the advantages of using sodium citrate as the setting regulator to form DCPD cements, and polymer coating, which can be used for the controlled release of biological cues.
